# Multiplex Analysis Platform for Endocrine Disruption Prediction Using Zebrafish

**DOI:** 10.3390/ijms20071739

**Published:** 2019-04-08

**Authors:** Sergio Jarque, Jone Ibarra, Maria Rubio-Brotons, Jessica García-Fernández, Javier Terriente

**Affiliations:** ZeClinics SL, Carretera de Can Ruti, Camí de les Escoles, s/n, Edificio IGTP Muntanya, 08916 Badalona, Barcelona, Spain; jone.ibarra@zeclinics.com (J.I.); maria.rubio@zeclinics.com (M.R.-B.); jessica.garcia@zeclinics.com (J.G.-F.)

**Keywords:** endocrine disruption, zebrafish, transcriptomics, estrogens, androgens, thyroid

## Abstract

Small fish are an excellent experimental model to screen endocrine-disrupting compounds, but current fish-based assays to detect endocrine disruption have not been standardized yet, meaning that there is not consensus on endpoints and biomarkers to be measured. Moreover, exposure conditions may vary depending on the species used as the experimental model and the endocrine pathway evaluated. At present, a battery of a wide range of assays is usually needed for the complete assessment of endocrine activities. With the aim of providing a simple, robust, and fast assay to assess endocrine-disrupting potencies for the three major endocrine axes, i.e., estrogens, androgens, and thyroid, we propose the use of a panel of eight gene expression biomarkers in zebrafish larvae. This includes brain aromatase (cyp19a1b) and vitellogenin 1 (vtg1) for estrogens, cytosolic sulfotransferase 2 family 2 (sult2st3) and cytochrome P450 2k22 (cyp2k22) for androgens, and thyroid peroxidase (tpo), transthyretin (ttr), thyroid receptor α (trα), and iodothyronine deiodinase 2 (dio2) for thyroid metabolism. All of them were selected according to their responses after exposure to the natural ligands 17β-estradiol, testosterone, and 3,3′,5-triiodo-L-thyronine (T3), respectively, and subsequently validated using compounds reported as endocrine disruptors in previous studies. Cross-talk effects were also evaluated for all compounds.

## 1. Introduction

Acute and long-term exposure of living organisms to environmental chemicals have been related to a wide range of physiological alterations and adverse health outcomes, including developmental toxicity, cancer, reduced fertility, obesity, diabetes, metabolic syndrome, and neurological disorders [1,2,3]. This results in evident risks to the environment and human health. Although most mechanisms of action remain to be elucidated, there is growing evidence that some of these effects may be due to alterations of the endocrine system (reviewed by [4,5,6]). As a consequence, the scientific community, together with international health and research organizations (e.g., World Health Organization, (WHO); Organisation for Economic Co-operation and Development, (OECD); Environmental Protection Agency, (EPA)), have placed focus on the identification, assessment of the potencies, and evaluation of the effects associated with the exposure of the so-called endocrine-disrupting compounds (EDCs), namely chemical substances that interact or interfere with one or more components of the endocrine system, potentially leading to harmful effects on human or animal health [7]. 

Several analytical tools have been developed for the detection of EDCs in different natural matrices [8,9,10]. Chemistry-based tools are sensitive and valid for detecting single compounds or compound families, but cannot measure biological activities or potentially harmful effects. In vitro tools are simple, inexpensive, and scalable to high-throughput platforms, but observations may have low relevance for vertebrate species. Experimentation in adult (vertebrate) animals may overcome most of those disadvantages, but it is expensive, laborious, time-consuming, low-throughput, and represents ethical issues, such as the incompatibility with the 3R principles (replacement, reduction, and refinement in animal testing).

Small fish, i.e., medaka (*Oryzias latipes*), zebrafish (*Danio rerio*), three-spined stickleback (*Gasterosteus aculeatus*), and fathead minnow (*Pimephales promelas*), are excellent experimental systems and suitable alternative models to animal testing for a wide range of applications, including assessment of endocrine-disrupting potencies [11,12], because of the large degree of conservation with higher vertebrates and the experimental advantages that they represent, namely transparency of embryos, reduced costs, large progenies, and adaptability to high-throughput contexts. However, current endocrine disruption tests based on small fish are scarce and typically not standardized, with only three methods under OECD guidelines [13,14,15]. All of them rely, among other parameters, on the quantification of plasma sex-specific proteins (vitellogenin and spiggin) after at least 21 days of exposure, belonging therefore to the category of animal testing. Within the early stages, most of the efforts so far have been focused on the validation and use of gene expression markers to assess estrogenicity [16] and the subsequent construction of specific transgenic lines combining estrogenic responsive promoters and fluorescent reporter genes [17,18]. Surprisingly, additional endocrine axes such as androgens and thyroid have been widely neglected. Indeed, it was not until recently that the scientific community has shown interest in the identification and validation of additional genes to be used as biomarkers for androgenic activity [19], with only one transgenic line in medaka available [20]. Thyroid disruptors are usually detected by measuring T3 and T4 levels [21], together with the measurement of mRNA levels for a relatively large number of genes within the pathway [22,23], making screenings tedious and unpractical in terms of throughput. Moreover, some of those genes, as is the case of *tg*, *tpo*, *slc5a5*, and *tshβ*, involved in TH synthesis, or *dio1*, *dio2*, and *dio3*, involved in TH activation/inactivation, might provide redundant information when applied for screening purposes. In this sense, the use of specific transgenic lines tries to offer a more suitable and specific high-throughput screening tool [24,25]. However, these lines are only valid to detect compounds directly interacting with the specific molecules designed to act as recognition elements, and given the complexity of the hypothalamus-pituitary-thyroid (HPT) axis [26], the use of various transgenic strains would be needed to assess fully thyroid-disrupting effects and avoid false negatives. In some cases, such as chemicals affecting thyroid transport or metabolism, this is not even possible since no transgenic line is available.

Consistent with the current needs to develop biosensors for the high-throughput screening of EDCs and reduce the number of different experimental models used, we propose a simple, robust, and fast transcriptomics-based assay to assess the estrogenic, androgenic, and thyroid-disrupting potencies of chemicals. The transcriptomic panel includes eight gene expression biomarkers, namely brain aromatase (*cyp19a1b*), vitellogenin 1 (*vtg1*), cytosolic sulfotransferase 2 family 2 (*sult2st3*), cytochrome P450 2k22 (*cyp2k22*), thyroid peroxidase (*tpo*), transthyretin (*ttr*), thyroid receptor α (*trα*), and iodothyronine deiodinase 2 (*dio2*), which were selected according to their robust responses after exposure to the natural ligands 17β-estradiol (E2), testosterone (TES), and 3,3′,5-triiodo-L-thyronine (T3). Validity of the method was further evaluated by testing specific antagonists, as well as a battery of compounds previously described as EDCs. Potential cross-reactions among the different endocrine axes were also considered for the three pathways.

## 2. Results and Discussion

### 2.1. Experimental Setup

**Figure 1:**
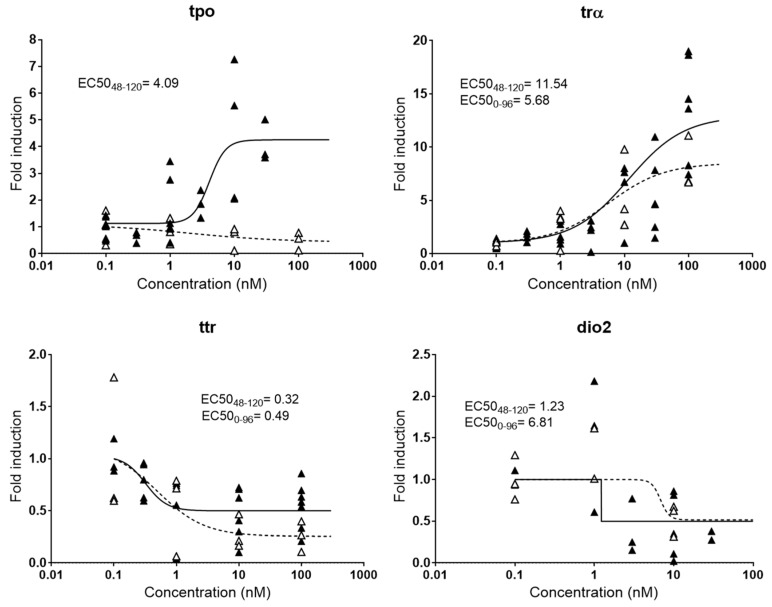
Comparative dose-response curves for four genes (*tpo*, *trα*, *ttr*, and *dio2*) involved in thyroid metabolism after exposure to T3 at different exposure windows. EC50s in nM are shown for responsive genes. Black triangles and continued line: exposure from 48–120 hpf; white triangles and dashed line: exposure from 0–96 hpf. Each data point indicates a biological replicate.

Small fish embryos have been widely used to assess endocrine disrupting potencies of chemicals, but no consensus has been established yet regarding the best window of exposure to detect effectively endocrine disruption. In many studies, embryos are exposed from 0–96 hpf [17], 0–72 hpf [16], or even 0–48 hpf [27], although it is known that some endocrine axis are not fully functional or even developed at those early stages [28]. Therefore, responses detected in those studies may be partially or totally due to unspecific toxicity and not specific hormone imbalance, resulting in false positives and/or false negatives. Other experimental embryonic setups extended the exposure window from 0–120 hpf with the aim of obtaining more robust responses [23], although likely still facing problems of toxicity. Indeed, our experiments showed that the expression patterns of some thyroid genes change significantly depending on the time of exposure to T3, the natural thyroid ligand (Figure 1). Among the genes evaluated, *tpo* was the one showing more drastic differences, with a clear induction when embryos were exposed from 48–120 hpf and a slight decrease when exposed from 0–96 hpf. Immunostaining and transcriptional methods proved that important thyroid gland elements such as thyroglobulin and the NIS symporter are not expressed until between 33 and 40 hpf, and it is estimated that the gland is fully functional from 55 hpf onwards [28]. Since *tpo* is also a gene located in the thyroid gland, the lack of response at 96 hpf most probably reflected the absence of thyroid peroxidase function and/or developmental affectation of the tissue by the earlier exposure. Lower differences were observed for *dio2*, with a relatively mild increase in the EC50 _(0–96 hpf)_ compared to the EC50 _(48–120 hpf)_. *trα* and *ttr* presented very similar dose-response curves for both exposure windows. 

Consistent with our results and previous observations, our setup allows the normal development of embryos until 48 hpf to later expose to the drug of interest from 48–120 hpf, an exposure window particularly recommended when assessing androgen and thyroid disruptors [19,29]. Accordingly, our experimental design was divided into two phases: The first one was an acute toxicity test from 48–120 hpf based on the OECD FET test using five different concentrations separated by a dilution factor ×10 in which the highest concentration tested was determined by the water solubility of the compound [30]. This experiment allowed us to determine LC50, LOEC, and NOEC (Table S1); the toxicity values were subsequently used to design a second experiment in which zebrafish embryos were exposed to a narrower exposure window of 3–7 different concentrations in which NOEC was the upper concentration. This second experiment served to detect the potential endocrine-disrupting potencies for each compound and was compatible with the screening of compounds effective for the three different endocrine axes. 

### 2.2. Gene Biomarker Validation

**Figure 2:**
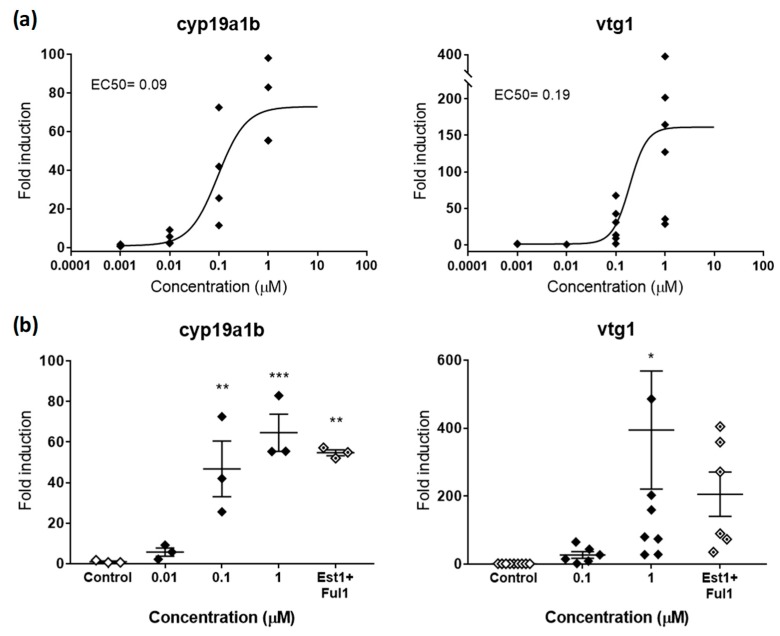
(**a**) Dose-response curve for *cyp19a1b* (left panel) and *vtg1* (right panel) in zebrafish embryos exposed to E2 from 48–120 hpf. EC50s in µM are shown for both genes. (**b**) Rescue effects of the antagonist FUL (1µM) on *cyp19a1b* (left panel) and *vtg1* (right panel) after co-exposure with E2 (1 µM). Each data point indicates a biological replicate. The mean ± SE is shown for each treatment. Results were significant when * *p* < 0.05, ** *p* < 0.01, *** *p* < 0.001 (one-way ANOVA plus Dunnett’s multiple comparison test).

**Figure 3:**
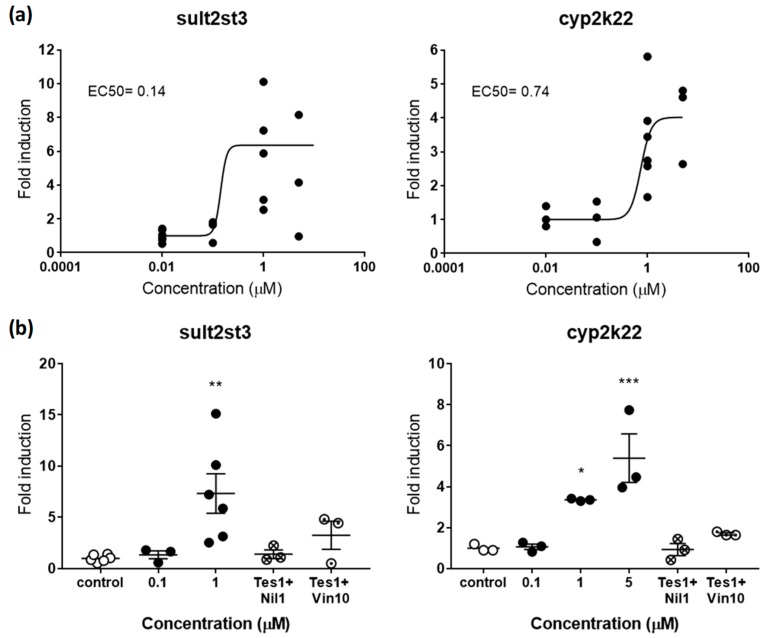
(**a**) Dose-response curve for *sult2st3* (left panel) and *cyp2k22* (right panel) in zebrafish embryos exposed to TES from 48–120 hpf. EC50s in µM are shown for both genes. (**b**) Rescue effects of the antagonists NIL (1 µM) and VIN (10 µM) on *sult2st3* (left panel) and *cyp2k22* (right panel) after co-exposure with TES (1 µM). Each data point indicates a biological replicate. The mean ± SE is shown for each treatment. Results were significant when * *p* < 0.05, ** *p* < 0.01, *** *p* < 0.001. 001 (one-way ANOVA plus Dunnett’s multiple comparison test).

**Figure 4:**
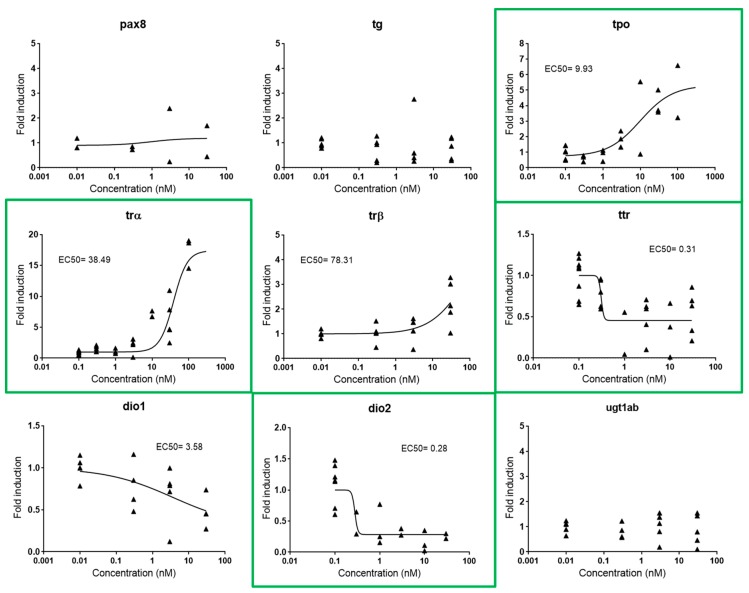
Dose-response curves for *pax8*, *tg*, *tpo*, *trα*, *trβ*, *ttr*, *dio1*, *dio2*, and *ugt1ab* in zebrafish embryos exposed to T3 from 48–120 hpf. EC50s are shown for all responsive genes. Each data point indicates a biological replicate. Biomarkers outlined in green were the biomarkers selected for further experiments.

Based on the available literature, our preliminary experiments considered a panel of 15 gene biomarkers, from which three were described to respond to estrogens (*cyp19a1b*, *vtg1*, *cyp19a*) [31], three to androgens (*sult2st3*, *cyp2k22*, *slco1f4*) [19], and nine to thyroid hormones (*pax8*, *tg*, *tpo*, *ttr*, *trα*, *trβ*, *dio1*, *dio2*, *ugt1ab*) [32,33]. However, *cyp19a* and *slco1f4* showed no response after exposure of embryos to E2 and TES, respectively, so they were not considered for further experiments (Figure S1). Regarding the thyroid axis, *pax8*, *tg*, *trβ*, *dio1*, and *ugt1ab* were also not further considered because of redundancy of information and less robustness compared to other genes (*tg*, *trβ*, and *dio1*) or because of a lack of response (*pax8* and *ugt1ab*) after exposure to the natural ligand T3. Considering these results, we designed a final panel including *cyp19a1b* and *vtg1* for (anti-)estrogenic potencies, *sult2st3* and *cyp2k22* for (anti-)androgenic potencies, and *tpo*, *trα*, *ttr* and *dio2* for thyroid potencies. All of them were modulated in a dose-response manner by their putative natural ligands (Figure 2a, Figure 3a, and Figure 4).

#### 2.2.1. Estrogenic and Antiestrogenic Potencies

*cyp19a1b* and *vtg1* were the two genes selected to detect chemicals with estrogenic activity. *cyp19a1b* encodes for brain aromatase, an enzyme that aromatizes testosterone (TES) to produce 17β-estradiol (E2) in the brain, while *vtg1* encodes for vitellogenin 1, a yolk precursor protein predominantly expressed in the liver and involved in the production of oocytes in adult females. Both genes, which are already active as soon as 24 hpf [27,34], present estrogen response elements in their promoter regions where the complex E2-estrogen receptor (ER) binds and regulates expression. In that sense, both proteins have been described as highly responsive when exposed to natural and synthetic estrogens and, therefore, considered good estrogenicity biomarkers. In our hands, E2 strongly upregulated *cyp19a1b* and *vtg1*, with maximum fold inductions (FI) compared to controls of 72.92 and 158.71, respectively. Effective potencies were very similar for both genes, with EC50s of 0.09 and 0.19 µM, respectively (Figure 2a). These potencies fit in the range of concentrations between 0.18 and 0.0024 µM previously reported in zebrafish embryos for both genes. Interestingly, *cyp19a1b* showed less induction, but a greater reproducibility among replicates (stdev*_cyp19a1b_* 21.18 vs. stdev*_vtg1_* 134.95). This is also something previously reported, and may be related to the nature of each gene. While *cyp19a1b* is expressed in the brain and involved in neurogenesis, a crucial process that needs a fine regulation already at early stages, *vtg1* is involved in reproduction and only naturally expressed in large amounts in mature females. Since reproduction is a process with a high intrinsic variability, it seems reasonable that *vtg1* reflected high variability also when induced in zebrafish embryos. Indeed, the variability observed in our study is equivalent to the variability reported not only in zebrafish adults (females and induced-males) [35], but also in other teleost species [31].

In order to validate the response of the gene biomarkers selected, we co-exposed embryos to effective concentrations of E2 with the classical antagonist fulvestrant (FUL) [36] and evaluated potential rescue effects. Estrogenic effects provoked by E2 (1 µM) were partially reduced by FUL (1 µM), decreasing FI from 64.54 to 57.17 for *cyp19a1b* and from 394.39 to 205.50 for *vtg1* (Figure 2b). Although significant, these rescue effects represented decreases of 15.24% and 47.89% compared to single exposures of E2, therefore not reaching control levels. These partial rescues were most probably due to the saturating concentrations of E2, a ligand with higher binding affinity to ER than FUL. This hypothesis is supported by the fact that concentrations 10 µM of FUL had no more rescue effects than those observed at 1 µM. In this regard, we suggest to evaluate the rescue effects of potential antagonists by using concentrations close to EC50 values rather than saturating concentrations, or replicate complete dose-response curves in co-exposure with antagonists to recognize potential EC50 shifts. However, other biological reasons behind the partial rescue effects cannot be ruled out. Indeed, several articles have shown that FUL was able to rescue estrogenic effects to a variable extent between 20% and 100%, depending on the estrogenic compound tested in co-exposure, with full rescues only observed for the less potent estrogens [17].

#### 2.2.2. Androgenic and Antiandrogenic Potencies

*sult2st3* and *cyp2k22* have been recently characterized as genes responsive to androgens and, therefore, potential gene biomarkers for detection of (anti-)androgenicity [19]. *sult2st3* encodes for a cytosolic sulfotransferase, an enzyme that catalyses the transfer of sulfonate groups from active sulfate to substrate compounds containing hydroxyl or amino groups suggested to be involved in androgen inactivation [37]. *cyp2k22* has been also related to androgenic steroids, most likely with a potential role in phase 1 metabolic reactions [19]. Recent toxicokinetic-toxicodynamic modelling studies support the hypothesis that expression of both genes is mediated by the androgen receptor (AR), although regulation of *cyp2k22* may include additional players. AR would be also the main regulator in this last case [19]. In agreement with these roles, embryos treated with TES induced the expression of sult2st3 and *cyp2k22* in a dose-response manner, with maximum average overexpression levels 7.33- and 4.01-times above controls, respectively (Figure 3a). EC50s were 0.14 µM for *sult2st3* and 0.74 for *cyp2k22*.

Similar to estrogens, validation of androgenic markers was tested by co-exposing TES with vinclozolin (VIN) or nilutamide (NIL), two well-known antiandrogens [38,39]. Overexpression induced by TES (1 µM) in *sult2st3* and *cyp2k22* was totally rescued, reaching control levels after co-exposure with 1 µM of NIL (Figure 3b). VIN (10 µM) was less effective, decreasing expression levels between 55.57 and 49.51% for both genes. Interestingly, TES 5 µM seemed to be a more effective concentration than 1 µM, at least for *cyp2k22*, while rescue effects were tested using TES 1 µM. This less potent concentration most probably facilitated drug competition and supports the idea that concentrations closer to EC50 values may be more suitable to identify antagonism.

#### 2.2.3. Thyroid

Thyroid metabolism is under the control of the HPT axis, a complex multi-loop feedback pathway that includes different processes in which the thyroid hormone (TH) is the major regulatory factor [26]. This complexity makes it difficult to identify single gene biomarkers covering the entire axis. Consequently, we propose the use of one gene representative for each of the four major thyroid processes within the pathway, i.e., synthesis, transport, signaling, and activation: *tpo* (thyroid peroxidase, an enzyme that oxidizes iodide ions to form the iodine atoms and produce TH from its precursor thyroglobulin), *trα* (thyroid receptor α, a nuclear receptor that mediates TH signaling), *ttr* (transthyretin, a transport protein), and *dio2* (iodothyronine deiodinase 2, an enzyme that activates TH) (Figure S2). These four genes appeared to be differently regulated by exogenous T3, *tpo* and *trα* being upregulated (FI 4.11 and 6.12, respectively) and *ttr* and *dio2* downregulated (FI 0.49 and 0.26, respectively). EC50s were 9.93, 38.49, 0.31, and 0.28 nM, respectively (Figure 4).

### 2.3. Screening of Compounds

**Figure 5:**
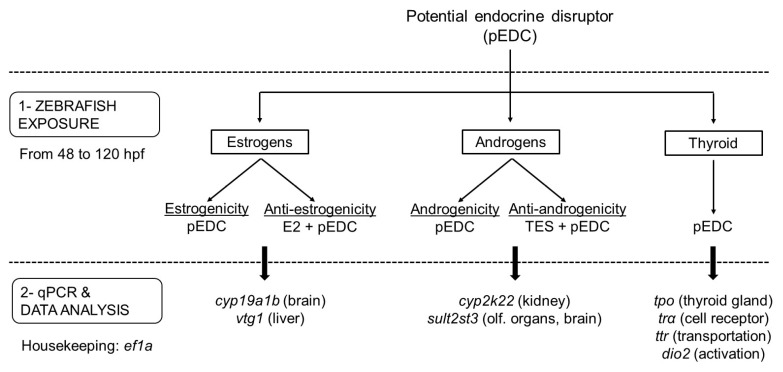
Flowchart representing the experimental sequence to be followed to test the different endocrine-disrupting activities related to a potential EDC (pEDC). After exposing embryos from 48–120 hpf (Step 1), gene expression is analyzed by qPCR (Step 2). The number of genes selected may depend on the pathway and organ of interest (specific targets are shown in brackets). Note that, in the case of not testing anti-endocrine responses, all three endocrine activities may be assessed in a single experiment by analyzing the expression of the eight genes of the panel. Evaluation of anti-estrogenicity and anti-androgenicity requires co-exposure with effective model compounds (e.g., E2 and TES, respectively).

**Figure 6:**
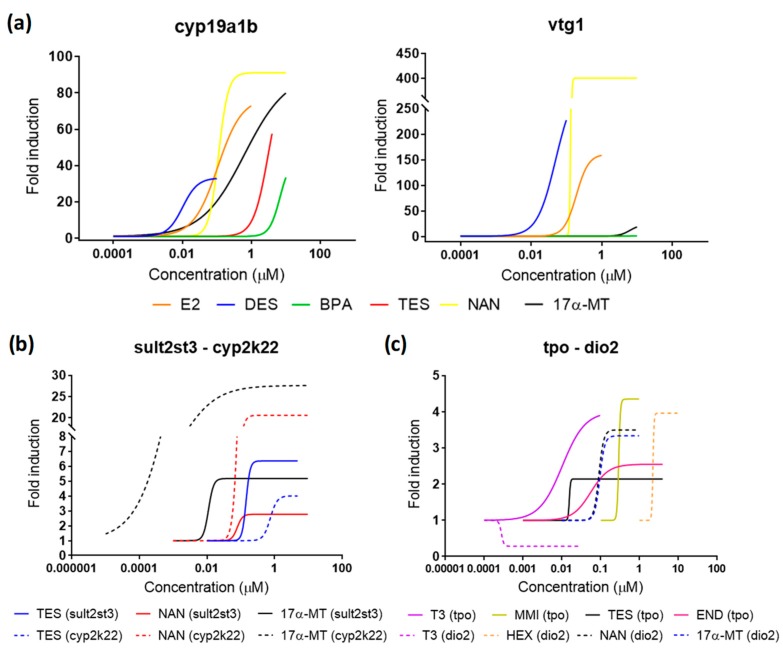
Dose-response curves for all compounds detected as EDCs in the present study. (**a**) Estrogenic compounds for *cyp19a1b* and *vtg1*. (**b**) Androgenic compounds for *sultst3* and *cyp2k22*. (**c**) Thyroid disrupting compounds for *tpo* and *dio2*. Continued and dashed lines in (**a**,**b**) differentiate the two gene biomarkers analyzed within the pathway. Data points for each replicate are not shown for better clarity.

**Figure 7:**
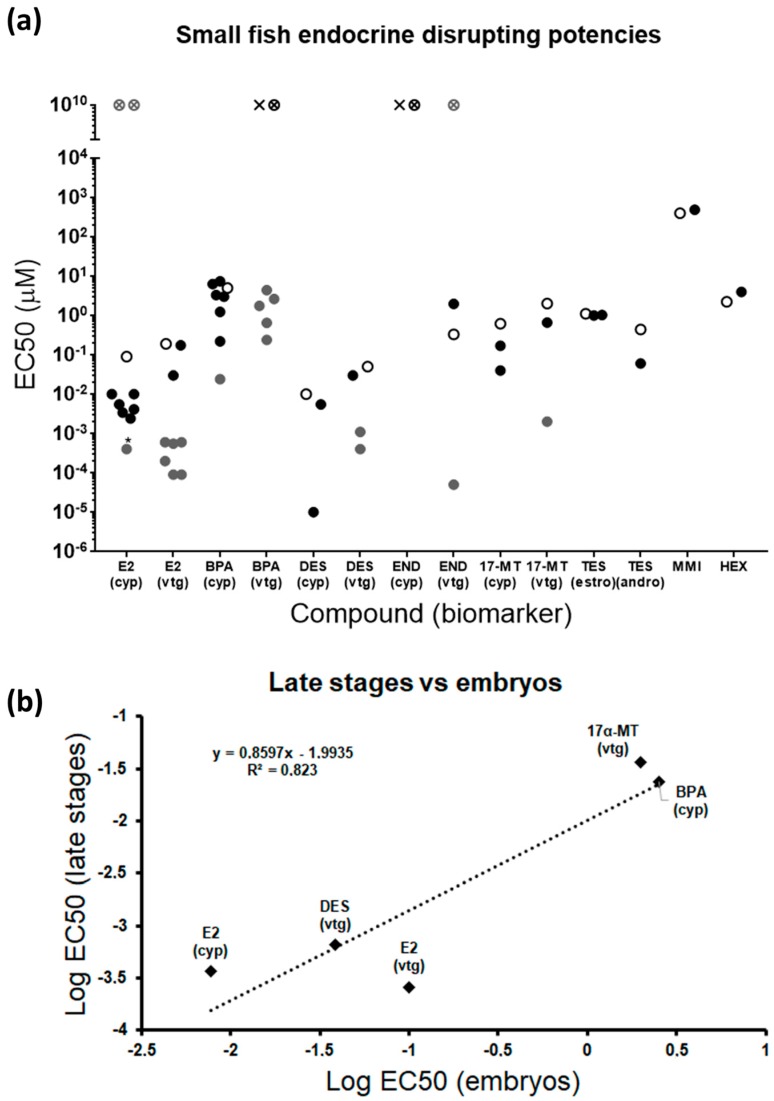
(**a**) Comparative graph showing EC50s from different endocrine-disrupting studies in small fish in early and late stages [9,16,17,18,19,23,27,29,34,35,40,42,59,60,61,62,63,65,66,67,68,69,70,71,72,73,74,75,76,77,78,79] Compounds not previously tested by others are excluded. Details for each study are shown in Table S4. Each data point represents the EC50 from each study. Black dots: studies performed in zebrafish embryos; white dots: this study; grey dots: studies performed in juveniles or adults (including zebrafish, medaka, three-spined stickleback, and fathead minnow); white crossed dot: compound with no effect in this study; cross: compound with no effect in other embryo studies; grey crossed dot: compound with no effect in juveniles or adults; *effects only observed in males; (estro) and (andro) denote that, because EC50s were similar, both biomarkers were considered together. (**b**) Correlation between endocrine-disrupting potencies in early and late stages. Only compounds and biomarkers tested and detected as positive in both developmental stages were included. END was not included in the comparison because contradictory results were found for adults in the literature. Each data point represents the average of potencies in embryos and adults.

Embryonic exposition with endocrine natural ligands allowed selecting the most appropriate incubation time and relevant biomarkers to set up a robust experimental flowchart (Figure 5). In order to further validate this procedure, we tested its robustness by testing chemicals previously described as EDCs. The list of tested chemicals included diethylstilbestrol (DES; a synthetic drug), bisphenol A (BPA; a chemical used in a wide range of industrial products and applications), and endosulfan (END; a pesticide) as estrogenic compounds, nandrolone (NAN; a synthetic drug) and 17α-methyltestosterone (17α-MT; a synthetic drug) as androgenic compounds, and methimazole (MMI; an antithyroid drug) and hexaconazole (HEX; fungicide) as thyroid disruptors. All of them were positively identified as EDCs in previous publications and our tests [17,19,23,25] (Figure 6, Table 1). Interestingly, specificities were detected for some compounds. Consistent with its use and previous experimental records, DES was the most potent compound in the group of estrogens, with an EC50 for both estrogenic biomarkers approximately one order of magnitude lower than E2. Furthermore, the fact that BPA and END altered only one of the two estrogenic biomarkers was remarkable, indicating different regulation between brain steroidogenesis and estrogens-mediated reproduction processes. BPA induced overexpression of *cyp19a1b*, with an EC50 of 4.99 μM, but did not affect neither positively or negatively the expression of *vtg1*. Estrogenic activity of BPA has been well documented in different species and life stages, including fish embryos and adults (Table S4), but its mode of action is still being debated. Acting on multiple steroid hormone receptors as agonist or antagonist [40], disrupting effects seem to be cell and tissue specific, as well as variable according to the stage of development. Indeed, evidence points out brain as the main target organ of BPA during embryonic stages [41]. Experiments in fish embryos using different setups showed that BPA upregulates *cyp19a1b* in a dose-response manner, with EC50s in the range between 1.23 and 7.4 µM, while *vtg1* seems to remain invariable, at least until 168 hpf [34] (Figure 7a, Table S4). This pattern is significantly changed in juveniles and adults, where BPA affects both genes in a similar extent [38]. END also showed a different expression profile depending on the gene, not affecting expression of *cyp19a1b*, but downregulating *vtg1* expression more than 50% (FI of 0.35) compared to controls for the most effective concentration (4 µM). In support of our results, END was previously identified as a non-estrogenic compound in transgenic embryos (*cyp19a1b*) [17] and estrogenic after 21 days of exposure in zebrafish adults (*vtg*) [42]. Nonetheless, *vtg* was induced, and not repressed, in that last study, partially contradicting our results. END is widely recognized as an endocrine disruptor, but the mechanisms of action have not been clarified yet. In vitro assays pointed out the ability of END to bind to ER in ER-transfected yeast and HeLa cells [43]. On the other hand, this pesticide was not able to induce vitellogenesis at concentrations up to 100 µM in carp hepatocytes, but to reduce effects of E2 after co-exposure [44]. In line with this repressive role in reproduction, END also inhibited oocyte maturation in zebrafish [45] and drastically reduced plasma vitellogenin levels in catfish [46], and no induction was observed in *vtg* expression after 21 days of exposure in sheepshead minnow [47]. Altogether, it is difficult to establish a conclusive estrogenic profile. It seems clear that END is not able to trigger estrogenic responses in brain. By contrast, there is sufficient evidence to include END as a potential endocrine disruptor in terms of reproduction. In addition to their specific profiles, both cases, BPA and END, point out the importance of including *cyp19a1b* and *vtg1* for the correct assessment of estrogenicity, as considering only one of them could result in false negatives.

The two model tested androgens induced the expression of both androgenic biomarkers *sult2st3* and *cyp2k22* (Figure 6, Table 1). 17α-MT and NAN showed very similar *sult2st3* induction potencies to that obtained for TES, with EC50s ranging from 0.10–0.14 µM and maximum FI from 4.64–8.97. More differences were observed in *cyp2k22* expression, 17α-MT being the most potent androgen, with an EC50 one order of magnitude lower than TES. TES was also the androgen with the lowest maximum FI, approximately three-times lower than those obtained for NAN and 17α-MT. TES is a natural ligand for AR, but not the predominant ligand in fish, which, unlike humans, has 11-ketosterone as the main androgen [48]. This less prevailing role may explain why FIs for TES were, in some cases, lower than those observed for other synthetic androgens. Since the effects on *sult2st3* and *cyp2k22* have not been previously assessed for NAN and 17α-MT, the results cannot be compared for this endpoint. Nevertheless, 17α-MT was also shown to be more potent than TES when assessing estrogenic cross-talk effects, showing lower EC50 or higher FI [17] (Figure 6a, Table 1; see Section 2.4. for more details). 

MMI is a well-known goitrogen, i.e., a compound that affects the synthesis of TH, used to treat hyperthyroidism. Transcriptomics and the use of tg(tg:mCherry) transgenic zebrafish demonstrated clear dose-response curves in four genes involved in TH synthesis (*tg*, *tpo*, *slc5a5*, and *tshβ*) after exposure of zebrafish embryos to a concentration series of MMI [25,29]. Experimental setups in both studies were comparable to the one presented here, using exposure windows from 48–120 hpf to avoid interference other than thyroid disruption in all cases. EC50 for *tpo* mRNA induction was 487 µM, while the mCherry signal was between 279 and 551 µM. In our study, MMI showed an EC50 equal to 397 µM, supporting those previous results and proving the reproducibility of the assay. None of the other three remaining thyroid markers analyzed were affected. HEX has been shown to decrease T4 and increase T3 contents, as well as to modulate the expression of several genes within the HPT axis, notably *ttr*, *trα*, and *dio2* [23]. Although our study also identified HEX as a thyroid disruptor, we could only replicate robust dose-responses for *dio2*, while *ttr* and *trα* remained unaltered. Since experimental setups from both studies were identical, with the only exception of the gene used as reference (*β-Actin* vs. *ef1a*) and the exposure window (0–120 hpf vs. 48–120 hpf), differences may be most probably attributed to the latter (see Section 2.1).

### 2.4. Cross-Talk Effects

Cross-talk effects among different endocrine axes have been widely reported [22,49,50] and became apparent also in our study (Table 1). Androgenic compounds were able to trigger expression of both estrogenic markers, with effective potencies, at least one order of magnitude higher than the ones obtained for E2. *cyp19a1b* was, in general, more sensitive than *vtg1* to androgens, as was disclosed by the higher FI after TES and 17α-MT treatments. Estrogenicity related to androgens was already documented in a previous study [17], and it is consistent with the role that androgens play as steroidogenic precursors of E2. The last step in the synthesis of E2 consists of the aromatization of TES by the enzyme aromatase, meaning that high concentrations of TES also result in increases of E2. As was observed for 17α-MT, other androgens may also undergo aromatization and trigger this process [51]. Moreover, some androgenic compounds are able to bind to the ER and, therefore, exert actions similar to E2 [52], including modulation of vitellogenin synthesis [53,54]. The potency order for *cyp19a1b* was DES > E2 > NAN > 17α-MT > TES > BPA. The same order was obtained by Brion et al. in screening experiments performed from 0–120 hpf using zebrafish embryos from the fluorescent transgenic line cyp19a1b-GFP [17], with the only exception being NAN, a chemical not tested in that study. It is important to remark that cyp19a1b-GFP embryos were on average one order of magnitude more sensitive than our assay. It is not clear whether sensitivity was increased because of the use of a different exposure windows (0–120 vs. 48–120 hpf), which could reflect unspecific effects other than estrogenic disruption, or because of a genuinely higher sensitivity related to the experimental model. Since EC50s reported from other studies using exposing windows starting from 0 hpf were comparable to the EC50s presented here, the latter seems more likely. The effective potency disclosed a slightly different order when quantifying *vtg1*, NAN being less effective and END showing anti-estrogenic effects: DES > E2 > END (antiestrogen) > TES > NAN > 17α-MT. To our knowledge, this report is the first evidence of the capacity of NAN to regulate strongly both steroidogenesis and vitellogenesis in fish embryos. Although less present than other androgens, NAN and some derivatives have been detected in river waters in concentrations up to 0.0002 µM [55]. Even though concentrations able to induce estrogenicity in our experiments were approximately three orders of magnitude higher, it is expected that, if also active, NAN effective concentrations for adults could be lower than effective concentrations reported for embryos, as occurs for other estrogenic disruptors (Figure 7a). This points out NAN as a chemical of potential concern in those environments where human activities related to this compound may be more present. TES, NAN, and 17α-MT also upregulated in a dose-response manner the expression of at least one thyroid marker (Table 1), proving that androgens can modulate the HPT axis.

Unlike androgens, estrogens were not able to induce expression in androgenic and thyroid pathways in the range of concentrations tested in our study. In fact, the only compound that altered in a consistent concentration-response relationship the expression of *tpo* was the antiestrogen END. However, since dilution factors for estrogens were optimized to detect effects on the parental pathway, it is possible that the lack of response may be due to the relatively low concentrations tested. It is interesting to note that BPA was recognized as a thyroid antagonist in cell-based assays [56] and described to alter some genes within the HPT axis in vivo [57,58]. However, results in vivo have to be taken cautiously since embryos were exposed from 0–72 hpf in both cases, an exposure window that may interfere with the development of the thyroid gland rather than affecting thyroid signaling. Indeed, experiments in a zebrafish transgenic line support that BPA’s actions on the HPT axis are significantly different depending on the exposure window, the embryos exposed at stages earlier than 48 hpf being more responsive than those exposed from 48–120 hpf [24].

Finally, none of the thyroid compounds were able to modulate the estrogenic or androgenic markers (Table 1), reflecting the selective nature of these compounds in the HPT axis and the limited action on the other pathways.

### 2.5. Zebrafish Embryos vs. Juveniles and Adults

Adult-based assays are considered more suitable than embryo-based assays for screening endocrine disruptors because of the possibility that late stages give for evaluating additional endpoints such as sex ratios, gonadal-somatic index, and body weight [13,14,59]. Despite this advantage, comparative studies between embryos and juveniles or adults are scarce and not conclusive regarding the suitability of using early developmental stages. In fact, embryos from small fish, particularly zebrafish, rather than adults have been used during the last decade to develop and optimize high-throughput screening methods for detecting EDCs [17,21,25]. Those new approaches mainly based on transcriptomics or transgenesis allow us now the short-term screening of large amounts of compounds and the fast assessment of disrupting potencies. Whether the results obtained by these methods are representative or not for later stages remains unsolved, and since the list of chemicals tested in adults is very limited, important cross-validation efforts would be required to properly answer this question. Because almost no experiments for thyroid disruptors exist in the late stages, this effort should be even higher for this group of compounds. Nevertheless, the data available, including those originated in the current study, can be used to compare effects among treatments and infer suitability of embryos for predicting disrupting actions. 

A mandatory feature to accept an experimental method is a reasonable reproducibility among experiments. In that sense, EC50s obtained from different studies testing the same chemicals in small fish embryos disclosed mostly very similar endocrine potencies, even though the exposure window and the endpoint measured were not identical in all cases (Figure 7a, Table S4). In fact, the variability of some compounds was higher because of the higher sensitivity associated with the transgenic line used in one specific study [17]. Importantly, the same compound was detected as positive or negative in all embryo assays. Differences in biomarker induction between embryos and late stages were evident for some compounds, particularly estrogenic disruptors, but no regular regulatory pattern was identified. E2 induces both estrogenic biomarkers in fish embryos, but effects in adults appear to be restricted to males when applied to *cyp19a1b* (Figure 7a) [60,61,62]. The inverse pattern is obtained for BPA, not regulating embryonic *vtg*, but severely increasing expression in adults [34]. DES and 17α-MT indistinctly modulate the expression of both biomarkers in embryos and adults. END was not able to induce *cyp19a1b* in embryos, and disrupting actions are unclear in adults [42,47]. Taken together, these variable responses reflect that estrogenic compounds can selectively modulate gene expression through different specific modes of action and that the sensitivity of molecular targets depends on the developmental stage. In this regard, chemicals acting on steroidogenesis may be more effective in early stages due to the importance of this process during development, while chemicals exclusively affecting reproduction would be more prone to alter expression in late stages, when reproductive structures are fully developed. In addition, reproductive effects may be gender-dependent, inducing expression in embryos and males [63], but repression in females [64]. This specific behavior cannot be ruled out for androgens and thyroid disruptors, although the lack of studies for both pathways in adults does not allow us to derive relevant conclusions. 

All compounds previously identified as EDC in adults and tested here were detected as positive in larvae for, at least, one biomarker within their parental endocrine pathway, proving the representativeness of the larval model and the robustness of the gene biomarkers selected (Figure 7a). Complementarity of the genes from the transcriptomic panel was also evident by the fact that some compounds specifically altered only one of their putative genes, notably estrogenic and thyroid disruptors. Therefore, it is strongly suggested to test all the genes assigned to each endocrine pathway to avoid false negatives. Embryonic endocrine-disrupting potencies calculated as the mean of EC50s for each compound and biomarker obtained in this and previous studies (Table S4) disclosed the order T3 > DES > E2 > 17α-MT > TES (androgenic biomarkers) > NAN > END > TES (estrogenic biomarkers) > BPA > HEX > MMI. This is the same order obtained for adults, with the only exception being DES, which appeared to be less potent than E2 in late stages. Correlation studies, although limited by the few compounds tested in adults, showed that fish embryos maintain a close potency relationship with later stages, adults and juveniles being between one and two orders of magnitude more sensitive for the same compound (Figure 7b, Table S4). These results point out zebrafish embryos as a valid prediction tool for the detection of EDCs. Nevertheless, evaluating a more extensive list of compounds should be considered in both developmental stages to increase the experimental significance.

## 3. Materials and Methods

### 3.1. Chemicals

All chemicals were purchased from Sigma-Aldrich: bisphenol A (BPA; purity ≥99%), diethylstilbestrol (DES; purity ≥99%), endosulfan (END; analytical standard grade), 17β-estradiol (E2; purity ≥98%), fulvestrant (FUL; purity ≥98%), hexaconazole (HEX; analytical standard grade), methimazole (MMI; analytical standard grade), 17α-methyltestosterone (17-αMT; purity ≥97.0%), nandrolone (NAN; analytical standard grade), nilutamide (NIL; solid), testosterone (TES; purity ≥99%), 3,3′,5-triiodo-l-thyronine (T3; purity ≥95%), vinclozolin (VIN; analytical standard grade). Stock solutions for all chemicals were prepared in dimethyl sulfoxide (DMSO), with the only exception being MMI, which was prepared in E3 media (Table S1).

### 3.2. Zebrafish Maintenance and Breeding

Adult wild-type AB strain fish were cultured at 28.5 ± 1 °C in a 14:10 h light:dark cycle in a recirculating tank system. Zebrafish embryos were obtained by mating adult fish through standard methods [80].

### 3.3. Toxicity Tests

Twenty embryos per treatment were exposed to 5 concentrations of compound of interest in a 10× dilution factor from 48–120 hpf, which is the experimental time in which EDCs will be screened posteriorly. Embryos were exposed from 48–120 hpf rather than 0–96 hpf or 0–120 hpf to avoid unspecific toxicity outside of the EDC testing period, which have been chosen on the basis that endocrine pathways, e.g., thyroid metabolism, are not fully functional until 40–55 hpf in zebrafish embryos [81]. Stock solutions of chemicals to be tested were freshly prepared in exposure medium. The highest concentration for each compound was selected according to its water solubility (Table S1). Exposures were performed in glass vessels to assure maximum bioavailability for hydrophobic compounds, and medium was not exchanged during the experiment.

### 3.4. EDCs Exposure Tests

Twenty embryos per treatment and replicate were exposed to 3–7 different concentrations of chemical in glass. The range of concentrations for each compound was selected by considering NOECs obtained in the toxicity tests (highest concentration tested) and the effective concentrations reported previously in similar studies [17]. Between 3 and 6 biological replicates were performed per compound and condition in, at least, two independent experiments. Antagonistic effects were evaluated by co-exposing zebrafish embryos to effective concentrations of natural ligand and effective concentrations of well-known antagonists.

### 3.5. Gene Expression Analysis by qPCR

At 120 hpf, 20 embryos per condition and replicate were pooled and homogenized in TRIreagent and total RNA extracted following the manufacturer’s protocol. Total RNA concentration was estimated by spectrophotometric absorption at 260 nm in a NanoDrop Spectrophotometer ND-1000 (NanoDrop Technologies; Wilmington, DE, USA). One hundred nanograms of RNA were retrotranscribed to cDNA by reverse transcriptase (Superscript III RT-Enzyme, Invitrogen, Carlsbad, CA, USA) and stored at −20 °C. Aliquots 10-times diluted of cDNA were used to quantify the amounts of specific transcripts in a Lightcycler^®^ 480 system (Roche, Basel, Switzerland) by the SYBR GREEN method. Annealing temperature used in amplification reactions was 60 °C in all cases. Genes considered as potential gene expression biomarkers were aromatase (*cyp19a1*), brain aromatase (*cyp19a1b*), and vitellogenin 1 (*vtg1*) for estrogens, solute carrier organic anion transporter family member 1F4 (*slco1f4*), cytosolic sulfotransferase 2 family 2 (*sult2st3*), and cytochrome P450 2k22 (*cyp2k22*) for androgens and thyroglobulin (*tg*), thyroid peroxidase (*tpo*), transthyretin (*ttr*) thyroid receptor α (*trα*), thyroid receptor β (*trβ*), iodothyronine deiodinase 1 (*dio1*), iodothyronine deiodinase 2 (*dio2*), and UDP glucuronosyltransferase 1 family a, b (*ugt1ab*) for thyroid metabolism. Accession numbers, primer sequences, and efficiencies for each gene are shown in Table S2. Relative expression levels were quantified using the ΔΔCt method [82] and ef1a as reference gene [83] (Table S3).

### 3.6. Statistical Analysis

Dose-response curves were calculated with a four-parametric log-logistic model using GraphPad Prism v 7.04 (GraphPad Software Inc., La Jolla, CA, USA), fixing the minimum effect levels in upregulated biomarkers and the maximum effect levels in downregulated ones, respectively, to 1. When the solubility of compounds did not allow reaching the curves’ plateaus, we fixed the maximum effects levels using the mean obtained in the most effective concentration tested.

Differences between treatments were calculated using one-way ANOVA plus Dunnett’s multiple comparison test.

### 3.7. Ethical Study Approval

All protocols have been approved by the Institutional Animal Care and Use Ethic Committee (Barcelona Biomedical Research Park [PRBB]–IACUEC) and implemented according to national and European regulations. All experiments were carried out in accordance with the principles of the 3Rs. Specifically, experiments have been performed up to 120 hpf; a developmental stage in which zebrafish embryos are still not considered animals by European laws (Directive 2010/63/EU). Hence, all protocols refer to the approved protocol “Zebrafish (Danio rerio) breeding for colony maintenance and transgenic colony creation” (CEA-OH/9421/2).

## 4. Conclusions

In the present study, we have developed and optimized a zebrafish embryo-based multiplex method for the parallel detection of estrogenic, androgenic, and thyroid disruptors. The three endocrine activities were detected using specific gene biomarkers validated after exposure to their respective natural ligands and chemicals previously reported as EDCs, sharing identical experimental setups, allowing simultaneous assessment of estrogenicity, androgenicity, and thyroid disruption for a given chemical. This validation study has allowed us to identify the most responsive genes, which allows reducing the workload and making the assay more compatible with high-throughput screenings. Comparative studies show that our approach is valid, obtaining effective potencies similar to those already reported for small fish embryos in the literature. In addition, we provided dose-response curves for some chemicals (e.g., nandrolone and 17α-MT) not tested so far that may be of environmental concern.

Importantly, our experiments have demonstrated that, at least, some genes involved in the thyroid axis show different expression response depending on the exposure window and that exposures initiated at 0 hpf may not be appropriate to reliably detect thyroid disruptors. This is consistent with the developmental pattern described for the thyroid gland in zebrafish and may invalidate some of the results previously published by others.

Although in general less sensitive than adult-based methods, the scarce data available in adults precludes making robust comparative studies between life stages. Nevertheless, all model compounds tested and previously reported as EDC in adults were successfully detected as positive in our assay, showing a good EC50 correlation. Moreover, specific modes of action were apparent for some compounds, e.g., BPA, selectively inducing one of the gene biomarkers.

Overall, our data confirm zebrafish larvae as a robust experimental model for predicting EDC activity in its three main axes. In that sense, our optimized multiplex transcriptomic platform represents a valuable asset for predicting such activity in an economic, reliable, high-throughput, and scalable manner and tries to contribute to the standardization of embryo-based assays for EDCs’ detection

## Figures and Tables

**Table 1:** Endocrine-disrupting effects of each tested compound on the estrogenic, androgenic, and thyroid axis.

Parental Pathway	Compound	Estrogen Axis	Androgen Axis	Thyroid Axis
Estrogens	E2	cyp19a1b (0.09) vtg 1 (0.19)		
	BPA	cyp19a1b (4.99)		
	END	vtg1 (0.33)		tpo (0.06)
	DES	cyp19a1b (0.01) vtg1 (0.05)		
Androgens	TES	cyp19a1b (1.11) vtg1 (1.46)	sult2st3 (0.14) cyp2k22 (0.74)	tpo (0.02)
	NAN	cyp19a1b (0.2) vtg1 (1.72)	sult2st3 (0.13) cyp2k22 (0.11)	dio2 (0.08)
	17α-MT	cyp19a1b (0.62) vtg1 (2)	sult2st3 (0.10) cyp2k22 (0.05)	dio2 (0.09)
Thyroid	T3		not evaluated	tpo (0.01) trα (0.038) ttr (0.0003) dio2 (0.0003)
	MMI			tpo (397)
	HEX		not evaluated	dio2 (2.22)

Green box: upregulation; red box: downregulation; orange box: up- and down-regulation depending on the gene; blue box: no effect; white box: not evaluated. EC50s (µM) for each gene are shown in brackets.

## Supplementary Materials

Supplementary materials can be found at https://www.mdpi.com/1422-0067/20/7/1739/s1.

## Abbreviations

EDC	endocrine disrupting compound
HPT	hypothalamus-pituitary-thyroid axis
TH	thyroid hormone
LC50	half maximal lethal concentration
EC50	half maximal effective concentration
LOEC	lowest observed effect concentration
NOEC	no observed effect concentration
ER	estrogen receptor
AR	androgen receptor
FI	fold induction
BPA	bisphenol A
pEDC	potential endocrine disrupting compound
DES	Diethylstilbestrol
END	Endosulfan
E2	17β-estradiol
FUL	Fulvestrant
HEX	Hexaconazole
MMI	Methimazole
17-αMT	17α-methyltestosterone
NAN	Nandrolone
NIL	Nilutamide
TES	Testosterone
T3	3,3′,5-triiodo-L-thyronine
VIN	Vinclozolin
DMSO	dimethyl sulfoxide
E3	control media

## References

[1] Le Magueresse-Battistoni B., Vidal H., Naville D. (2018). Environmental Pollutants and Metabolic Disorders: The Multi-Exposure Scenario of Life. Front. Endocrinol..

[2] Sfakianakis D.G., Renieri E., Kentouri M., Tsatsakis A.M. (2015). Effect of heavy metals on fish larvae deformities: A review. Environ. Res..

[3] Chen H., Wang C., Li H., Ma R., Yu Z., Li L., Xiang M., Chen X., Hua X., Yu Y. (2019). A review of toxicity induced by persistent organic pollutants (POPs) and endocrine-disrupting chemicals (EDCs) in the nematode Caenorhabditis elegans. J. Environ. Manag..

[4] De Coster S., van Larebeke N. (2012). Endocrine-disrupting chemicals: Associated disorders and mechanisms of action. J. Environ. Public Health.

[5] Matthiessen P., Wheeler J.R., Weltje L. (2018). A review of the evidence for endocrine disrupting effects of current-use chemicals on wildlife populations. Crit. Rev. Toxicol..

[6] Brown S.B., Adams B.A., Cyr D.G., Eales J.G. (2004). Contaminant effects on the teleost fish thyroid. Environ. Toxicol. Chem..

[7] Damstra T., Barlow S., Bergman A., Kavlock R., Van der Kraak G., WHO (2002). Global Assessment of the State-of-the-Science of Endocrine Disruptors.

[8] Jarque S., Bittner M., Blaha L., Hilscherova K. (2016). Yeast Biosensors for Detection of Environmental Pollutants: Current State and Limitations. Trends Biotechnol..

[9] Petersen K., Fetter E., Kah O., Brion F., Scholz S., Tollefsen K.E. (2013). Transgenic (cyp19a1b-GFP) zebrafish embryos as a tool for assessing combined effects of oestrogenic chemicals. Aquat. Toxicol..

[10] Paul K.B., Hedge J.M., Rotroff D.M., Hornung M.W., Crofton K.M., Simmons S.O. (2014). Development of a thyroperoxidase inhibition assay for high-throughput screening. Chem. Res. Toxicol..

[11] Ankley G.T., Johnson R.D. (2004). Small fish models for identifying and assessing the effects of endocrine-disrupting chemicals. ILAR J..

[12] Scholz S., Mayer I. (2008). Molecular biomarkers of endocrine disruption in small model fish. Mol. Cell. Endocrinol..

[13] OECD (2009). Test No. 230: 21-Day Fish Assay.

[14] OECD (2012). Test No. 229: Fish Short Term Reproduction Assay.

[15] OECD (2011). Test No. 234: Fish Sexual Development Test [Internet]. https://www.oecd-ilibrary.org/environment/test-no-220-enchytraeid-reproduction-test_9789264264472-en.

[16] Kishida M., McLellan M., Miranda J.A., Callard G.V. (2001). Estrogen and xenoestrogens upregulate the brain aromatase isoform (P450aromB) and perturb markers of early development in zebrafish (*Danio rerio*). Comp. Biochem. Physiol. B Biochem. Mol. Biol..

[17] Brion F., Le Page Y., Piccini B., Cardoso O., Tong S.-K., Chung B., Kah O. (2012). Screening Estrogenic Activities of Chemicals or Mixtures In Vivo Using Transgenic (cyp19a1b-GFP) Zebrafish Embryos. PLoS ONE.

[18] Green J.M., Metz J., Lee O., Trznadel M., Takesono A., Brown A.R., Owen S.F., Kudoh T., Tyler C.R. (2016). High-Content and Semi-Automated Quantification of Responses to Estrogenic Chemicals Using a Novel Translucent Transgenic Zebrafish. Environ. Sci. Technol..

[19] Fetter E., Smetanová S., Baldauf L., Lidzba A., Altenburger R., Schüttler A., Scholz S. (2015). Identification and Characterization of Androgen-Responsive Genes in Zebrafish Embryos. Environ. Sci. Technol..

[20] Sébillot A., Damdimopoulou P., Ogino Y., Spirhanzlova P., Miyagawa S., Du Pasquier D., Mouatassim N., Iguchi T., Lemkine G.F., Demeneix B.A. (2014). Rapid fluorescent detection of (anti)androgens with spiggin-gfp medaka. Environ. Sci. Technol..

[21] Thienpont B., Tingaud-Sequeira A., Prats E., Barata C., Babin P.J., Raldúa D. (2011). Zebrafish eleutheroembryos provide a suitable vertebrate model for screening chemicals that impair thyroid hormone synthesis. Environ. Sci. Technol..

[22] Liu C., Yu H., Zhang X. (2013). Zebrafish embryos/larvae for rapid determination of effects on hypothalamic-pituitary-thyroid (HPT) and hypothalamic-pituitary-interrenal (HPI) axis: mRNA expression. Chemosphere.

[23] Yu L., Chen M., Liu Y., Gui W., Zhu G. (2013). Thyroid endocrine disruption in zebrafish larvae following exposure to hexaconazole and tebuconazole. Aquat. Toxicol..

[24] Terrien X., Fini J.-B., Demeneix B.A., Schramm K.-W., Prunet P. (2011). Generation of fluorescent zebrafish to study endocrine disruption and potential crosstalk between thyroid hormone and corticosteroids. Aquat. Toxicol..

[25] Jarque S., Fetter E., Veneman W.J., Spaink H.P., Peravali R., Strähle U., Scholz S. (2018). An automated screening method for detecting compounds with goitrogenic activity using transgenic zebrafish embryos. PLoS ONE.

[26] Jarque S., Piña B. (2014). Deiodinases and thyroid metabolism disruption in teleost fish. Environ. Res..

[27] Lassiter C.S., Linney E. (2007). Embryonic Expression And Steroid Regulation of Brain Aromatase cyp19a1b in Zebrafish (*Danio rerio*). Zebrafish.

[28] Alt B., Reibe S., Feitosa N.M., Elsalini O.A., Wendl T., Rohr K.B. (2006). Analysis of origin and growth of the thyroid gland in zebrafish. Dev. Dyn..

[29] Fetter E., Baldauf L., Da Fonte D.F., Ortmann J., Scholz S. (2015). Comparative analysis of goitrogenic effects of phenylthiourea and methimazole in zebrafish embryos. Reprod. Toxicol..

[30] OECD (2013). Test No. 236: Fish Embryo Acute Toxicity (FET) Test.

[31] García-Reyero N., Raldúa D., Quirós L., Llaveria G., Cerdà J., Barceló D., Grimalt J.O., Piña B. (2004). Use of vitellogenin mRNA as a biomarker for endocrine disruption in feral and cultured fish. Anal. Bioanal. Chem..

[32] Yang M., Hu J., Li S., Ma Y., Gui W., Zhu G. (2016). Thyroid endocrine disruption of acetochlor on zebrafish (*Danio rerio*) larvae. J. Appl. Toxicol..

[33] Zhang D., Zhou E., Yang Z. (2017). Waterborne exposure to BPS causes thyroid endocrine disruption in zebrafish larvae. PLoS ONE.

[34] Chen M., Zhang J., Pang S., Wang C., Wang L., Sun Y., Song M., Liang Y. (2018). Evaluating estrogenic and anti-estrogenic effect of endocrine disrupting chemicals (EDCs) by zebrafish (*Danio rerio*) embryo-based vitellogenin 1 (vtg1) mRNA expression. Comp. Biochem. Physiol. Part C Toxicol. Pharmacol..

[35] Brian J.V., Harris C.A., Scholze M., Backhaus T., Booy P., Lamoree M., Pojana G., Jonkers N., Runnalls T., Bonfà A. (2005). Accurate Prediction of the Response of Freshwater Fish to a Mixture of Estrogenic Chemicals. Environ. Health Perspect..

[36] Hall J.M., Couse J.F., Korach K.S. (2001). The multifaceted mechanisms of estradiol and estrogen receptor signaling. J. Biol. Chem..

[37] Fent K., Siegenthaler P.F., Schmid A.A. (2018). Transcriptional effects of androstenedione and 17α-hydroxyprogesterone in zebrafish embryos. Aquat. Toxicol..

[38] Molina-Molina J.-M., Hillenweck A., Jouanin I., Zalko D., Cravedi J.-P., Fernández M.-F., Pillon A., Nicolas J.C., Olea N., Balaguer P. (2006). Steroid receptor profiling of vinclozolin and its primary metabolites. Toxicol. Appl. Pharmacol..

[39] Urushibara M., Ishioka J., Hyochi N., Kihara K., Hara S., Singh P., Isaacs J.T., Kageyama Y. (2007). Effects of steroidal and non-steroidal antiandrogens on wild-type and mutant androgen receptors. Prostate.

[40] Le Fol V., Aït-Aïssa S., Sonavane M., Porcher J.-M., Balaguer P., Cravedi J.-P., Zalko D., Brion F. (2017). In vitro and in vivo estrogenic activity of BPA, BPF and BPS in zebrafish-specific assays. Ecotoxicol. Environ. Saf..

[41] Nesan D., Sewell L.C., Kurrasch D.M. (2018). Opening the black box of endocrine disruption of brain development: Lessons from the characterization of Bisphenol A. Horm. Behav..

[42] Han Z., Jiao S., Kong D., Shan Z., Zhang X. (2011). Effects of β-endosulfan on the growth and reproduction of zebrafish (*Danio rerio*). Environ. Toxicol. Chem..

[43] Petit F., Le Goff P., Cravédi J.P., Valotaire Y., Pakdel F. (1997). Two complementary bioassays for screening the estrogenic potency of xenobiotics: Recombinant yeast for trout estrogen receptor and trout hepatocyte cultures. J. Mol. Endocrinol..

[44] Smeets J.M., van Holsteijn I., Giesy J.P., Seinen W., van den Berg M. (1999). Estrogenic potencies of several environmental pollutants, as determined by vitellogenin induction in a carp hepatocyte assay. Toxicol. Sci..

[45] Maskey E., Crotty H., Wooten T., Khan I.A. (2019). Disruption of oocyte maturation by selected environmental chemicals in zebrafish. Toxicol. In Vitro.

[46] Chakravorty S., Lal B., Singh T.P. (1992). Effect of endosulfan (thiodan) on vitellogenesis and its modulation by different hormones in the vitellogenic catfish Clarias batrachus. Toxicology.

[47] Hemmer M.J., Hemmer B.L., Bowman C.J., Kroll K.J., Folmar L.C., Marcovich D., Hoglund M.D., Denslow N.D. (2001). Effects of p-nonylphenol, methoxychlor, and endosulfan on vitellogenin induction and expression in sheepshead minnow (*Cyprinodon variegatus*). Environ. Toxicol. Chem..

[48] Fostier A., Jalabert B., Billard R., Breton B., Zohar Y., Hoar W.S., Randall D.J., Donaldson E.M. (1983). The Gonadal Steroids. Reproduction Endocrine Tissues and Hormones.

[49] Li W., Zhu L., Zha J., Wang Z. (2014). Effects of decabromodiphenyl ether (BDE-209) on mRNA transcription of thyroid hormone pathway and spermatogenesis associated genes in Chinese rare minnow (*Gobiocypris rarus*). Environ. Toxicol..

[50] Liu C., Zhang X., Deng J., Hecker M., Al-Khedhairy A., Giesy J.P., Zhou B. (2011). Effects of prochloraz or propylthiouracil on the cross-talk between the HPG, HPA, and HPT axes in zebrafish. Environ. Sci. Technol..

[51] Hornung M.W., Jensen K.M., Korte J.J., Kahl M.D., Durhan E.J., Denny J.S., Henry T.R., Ankley G.T. (2004). Mechanistic basis for estrogenic effects in fathead minnow (*Pimephales promelas*) following exposure to the androgen 17α-methyltestosterone: Conversion of 17α-methyltestosterone to 17α-methylestradiol. Aquat. Toxicol..

[52] Sirianni R., Capparelli C., Chimento A., Panza S., Catalano S., Lanzino M., Pezzi V., Andò S. (2012). Nandrolone and stanozolol upregulate aromatase expression and further increase IGF-I-dependent effects on MCF-7 breast cancer cell proliferation. Mol. Cell. Endocrinol..

[53] Kim B.-H., Takemura A., Kim S.J., Lee Y.-D. (2003). Vitellogenin synthesis via androgens in primary cultures of tilapia hepatocytes. Gen. Comp. Endocrinol..

[54] Mori T., Matsumoto H., Yokota H. (1998). Androgen-induced vitellogenin gene expression in primary cultures of rainbow trout hepatocytes. J. Steroid Biochem. Mol. Biol..

[55] Zhang J.-N., Ying G.-G., Yang Y.-Y., Liu W.-R., Liu S.-S., Chen J., Liu Y.S., Zhao J.L., Zhang Q.Q. (2018). Occurrence, fate and risk assessment of androgens in ten wastewater treatment plants and receiving rivers of South China. Chemosphere.

[56] Freitas J., Cano P., Craig-Veit C., Goodson M.L., Furlow J.D., Murk A.J. (2011). Detection of thyroid hormone receptor disruptors by a novel stable in vitro reporter gene assay. Toxicol. In Vitro.

[57] Lu L., Zhan T., Ma M., Xu C., Wang J., Zhang C., Liu W., Zhuang S. (2018). Thyroid Disruption by Bisphenol S Analogues via Thyroid Hormone Receptor β: In Vitro, in Vivo, and Molecular Dynamics Simulation Study. Environ. Sci. Technol..

[58] Berto-Júnior C., Santos-Silva A.P., Ferreira A.C.F., Graceli J.B., de Carvalho D.P., Soares P., Romeiro N.C., Miranda-Alves L. (2018). Unraveling molecular targets of bisphenol A and S in the thyroid gland. Environ. Sci. Pollut. Res..

[59] Dammann A.A., Shappell N.W., Bartell S.E., Schoenfuss H.L. (2011). Comparing biological effects and potencies of estrone and 17β-estradiol in mature fathead minnows, *Pimephales promelas*. Aquat. Toxicol..

[60] Hinfray N., Palluel O., Turies C., Cousin C., Porcher J.M., Brion F. (2006). Brain and gonadal aromatase as potential targets of endocrine disrupting chemicals in a model species, the zebrafish (*Danio rerio*). Environ. Toxicol..

[61] Kallivretaki E., Eggen R., Neuhauss S., Alberti M., Kausch U., Segner H. (2006). Aromatase in zebrafish: A potential target for endocrine disrupting chemicals. Mar. Environ. Res..

[62] Halm S., Pounds N., Maddix S., Rand-Weaver M., Sumpter J., Hutchinson T., Tyler C.R. (2002). Exposure to exogenous 17β-oestradiol disrupts P450aromB mRNA expression in the brain and gonad of adult fathead minnows (*Pimephales promelas*). Aquat. Toxicol..

[63] Pawlowski S., Sauer A., Shears J., Tyler C., Braunbeck T. (2004). Androgenic and estrogenic effects of the synthetic androgen 17α-methyltestosterone on sexual development and reproductive performance in the fathead minnow (*Pimephales promelas*) determined using the gonadal recrudescence assay. Aquat. Toxicol..

[64] Kang I.J., Yokota H., Oshima Y., Tsuruda Y., Shimasaki Y., Honjo T. (2008). The effects of methyltestosterone on the sexual development and reproduction of adult medaka (*Oryzias latipes*). Aquat. Toxicol..

[65] Chung E., Genco M.C., Megrelis L., Ruderman J.V. (2011). Effects of bisphenol A and triclocarban on brain-specific expression of aromatase in early zebrafish embryos. Proc. Natl. Acad. Sci. USA.

[66] Muncke J., Eggen R.I.L. (2006). Vitellogenin 1 mRNA as an early molecular biomarker for endocrine disruption in developing zebrafish (*Danio rerio*). Environ. Toxicol. Chem..

[67] Rose J., Holbech H., Lindholst C., Nørum U., Povlsen A., Korsgaard B., Bjerregaard P. (2002). Vitellogenin induction by 17beta-estradiol and 17alpha-ethinylestradiol in male zebrafish (*Danio rerio*). Comp. Biochem. Physiol. C Toxicol. Pharmacol..

[68] Van den Belt K., Berckmans P., Vangenechten C., Verheyen R., Witters H. (2004). Comparative study on the in vitro/in vivo estrogenic potencies of 17β-estradiol, estrone, 17α-ethynylestradiol and nonylphenol. Aquat. Toxicol..

[69] Parks L.G., Cheek A.O., Denslow N.D., Heppell S.A., McLachlan J.A., LeBlanc G.A., Sullivan C.V. (1999). Fathead minnow (*Pimephales promelas*) vitellogenin: Purification, characterization and quantitative immunoassay for the detection of estrogenic compounds. Comp. Biochem. Physiol. Part C Pharmacol. Toxicol. Endocrinol..

[70] Zeng Z., Shan T., Tong Y., Lam S.H., Gong Z. (2005). Development of Estrogen-Responsive Transgenic Medaka for Environmental Monitoring of Endocrine Disrupters. Environ. Sci. Technol..

[71] Saeed A., Hashmi I., Zare A., Mehrabani-Zeinabad M., Achari G., Habibi H.R. (2016). Efficacy of UV-C photolysis of bisphenol A on transcriptome alterations of genes in zebrafish embryos. J. Environ. Sci. Health Part A.

[72] Molina A., Abril N., Morales-Prieto N., Monterde J., Ayala N., Lora A., Moyano R. (2018). Hypothalamic-pituitary-ovarian axis perturbation in the basis of bisphenol A (BPA) reproductive toxicity in female zebrafish (*Danio rerio*). Ecotoxicol. Environ. Saf..

[73] Sohoni P.C.R.T., Tyler C.R., Hurd K., Caunter J., Hetheridge M., Williams T., Woods C., Evans M., Toy R., Gargas M. (2001). Reproductive Effects of Long-Term Exposure to Bisphenol A in the Fathead Minnow (*Pimephales promelas*). Environ. Sci. Technol..

[74] Zhong X., Xu Y., Liang Y., Liao T., Wang J. (2004). Vitellogenin in rare minnow (*Gobiocypris rarus*): Identification and induction by waterborne diethylstilbestrol. Comp. Biochem. Physiol. Part C Toxicol. Pharmacol..

[75] Yin P., Li Y.-W., Chen Q.-L., Liu Z.-H. (2017). Diethylstilbestrol, flutamide and their combination impaired the spermatogenesis of male adult zebrafish through disrupting HPG axis, meiosis and apoptosis. Aquat. Toxicol..

[76] Moon Y.-S., Jeon H.-J., Nam T.-H., Choi S.-D., Park B.-J., Ok Y.S., Lee S.E. (2016). Acute toxicity and gene responses induced by endosulfan in zebrafish (*Danio rerio*) embryos. Chem. Speciat. Bioavailab..

[77] Mouriec K., Gueguen M.-M., Manuel C., Percevault F., Thieulant M.-L., Pakdel F., Kah O. (2009). Androgens Upregulate cyp19a1b (Aromatase B) Gene Expression in the Brain of Zebrafish (*Danio rerio*) Through Estrogen Receptors1. Biol. Reprod..

[78] Trant J.M., Gavasso S., Ackers J., Chung B.C., Place A.R. (2001). Developmental expression of cytochrome P450 aromatase genes (CYP19a and CYP19b) in zebrafish fry (*Danio rerio*). J. Exp. Zool..

[79] Ankley G.T., Jensen K.M., Kahl M.D., Korte J.J., Makynen E.A. (2001). Description and evaluation of a short-term reproduction test with the fathead minnow (*Pimephales promelas*). Environ. Toxicol. Chem..

[80] Westerfield M. (2000). The Zebrafish Book; A Guide for the Laboratory Use of Zebrafish (Brachydanio rerio).

[81] Tonyushkina K.N., Shen M.-C., Ortiz-Toro T., Karlstrom R.O. (2014). Embryonic exposure to excess thyroid hormone causes thyrotrope cell death. J. Clin. Investig..

[82] Pfaffl M.W. (2001). A new mathematical model for relative quantification in real-time RT-PCR. Nucleic Acids Res..

[83] McCurley A.T., Callard G.V. (2008). Characterization of housekeeping genes in zebrafish: Male-female differences and effects of tissue type, developmental stage and chemical treatment. BMC Mol. Biol..

