# Supplementary material for: Multiplex Analysis Platform for Endocrine Disruption Prediction Using Zebrafish
Source: Int J Mol Sci. 2019 Apr 8;20(7):1739. doi: 10.3390/ijms20071739 (PMC6479714; doi:10.3390/ijms20071739)
Supplement: Supplementary file 1 [file ijms-20-01739-s001.pdf]

Multiplex analysis platform for Endocrine disruption prediction using zebrafish

Sergio Jarque[1], Jone Ibarra[1], María Rubio[1], Jessica García[1], Javier Terriente[1]

[1]ZeClinics SL, Carretera de Can Ruti, Camí de les Escoles, s/n, Edificio IGTP Muntanya, 08916, Badalona, Barcelona, Spain. sergio.jarque@zeclinics.com

Supplementary information

Table S1. Physicochemical properties, use, toxicity (LC50) and range of concentrations tested in endocrine disruption assays of each chemical. - indicates compounds in which mortality was not achieved

Name	CAS number	MW	Water solubility (mg/L)	Use	LC50 (μM)	Conc. range (μM)
Bisphenol A (BPA)	80-05-7	228.29	120.00	industrial product	41.56	0.10 - 10
Diethylstilbestrol (DES)	56-53-1	268.35	12.00	drug	-	0.001 - 0.1
Endosulfan (END)	115-29-7	406.93	0.45	pesticide	-	0.04 - 4
17β-estradiol (E2)	50-28-2	272.38	3.90	natural hormone	35.85	0.01 - 1
Fulvestrant (FUL)	129453-61-8	606.77	6.72	drug	-	1 - 10
Hexaconazole (HEX)	79983-71-4	314.21	17.00	fungicide	23.41	2 - 8
Methimazole (MMI)	60-56-0	114.17	277500.00	drug	15851.00	250 - 1000
17α-Methyltestosterone (17α-MT)	58-18-4	302.45	33.90	drug	90.21	0.001 - 10
Nandrolone (NAN)	434-22-0	274.40	3090.00	drug	497.10	0.01 - 10
Nilutamide (NIL)	63612-50-0	317.22	4.19	drug	32.85	1
Testosterone (TES)	58-22-0	288.42	23.40	natural hormone	77.83	0.01 - 5
3,3′,5-triiodo-L-thyronin (T3)	6893-02-3	650.97	3.96	natural hormone	0.006	0.0001 - 0.1
Vinclozolin (VIN)	50471-44-8	286.11	2.60	fungicide	-	10

Table S2. Primers sequences, amplicon lengths and amplification efficiencies for the genes analyzed in the study. – indicates that efficiencies were not calculated because of the low basal levels.

Gene	Accession number	Forward 5′-3′	Reverse 5′-3′	Efficiency (%)
ef1a	L47669.1	AGCAGCAGCTGAGGAGTGAT	CCGCATTTGTAGATCAGATGG	98
cyp19a1	NM_131154.3	TGGGTCGAATGCACAGATCC	GATCCGAACGGCTGGAAGAA	-
cyp19a1b	AF226619.1	TCGGCACGGCGTGCAACTAC	CATACCTATGCATTGCAGACC	93
vtg1	NM_001044897.3	CCTGCTCCATTTGACAGAACC	GTCCAGGATTTCCCTCAGT	93
sult2st3	NM_001078168.2	GACCACATCAAAAGCTGGCGAAAC	GTGCTGTTACTGACGACACGATCC	104
cyp2k22	NM_200235.1	CGTCAGACCAGCTGTGATGT	TGTCAGGTGTTTCCCACTCA	95
slco4f1	NM_001080666.2	GCCGTACCTTTCTTCGCTCTCAG	GGTCACTCCATTCTCTCCACACAC	-
tg	DQ278875.1	CTGGTCACCTGTGGTTGATG	TCCCTGAAGCTGCTCAAAAT	107
tpo	XM_021467270.1	CCAGCCAGACCTCGTTC	CGGAGATGAGCGGAAGAAG	110

pax8	AF072549.1	GAAGATCGCGGAGTACAAGC	CTGCACTTTAGTGCGGATGA	-
ttr	BC081488.1	CGGGTGGAGTTTGACACTTT	GCTCAGAAGGAGAGCCAGTG	94
trα	NM_131396.1	CTATGAACAGCACATCCGACAAGAG	CACACCACACACGGCTCATC	91
trβ	NM_131340.1	TGGGAGATGATACGGGTTGT	ATAGGTGCCGATCCAATGTC	99
dio1	BC076008.1	GTTCAAACAGCTTGTCAAGGACT	AGCAAGCCTCTCCTCCAAGTT	92
dio2	NM_212789.4	GCATAGGCAGTCGCTCATTT	TGTGGTCTCTCATCCAACCA	90
ugt1ab	NM_213422.2	CCACCAAGTCTTTCCGTGTT	GCAGTCCTTCACAGGCTTTC	85

Table S3. Stability of ef1a determined by Bestkeeper© software. Standard deviation below 1 is considered valid for a housekeeping gene. Red cells indicate standard deviations above 1 (target genes).

	ef1a (HK)	cyp19a1b	vtg1	sult2st3	cyp2k22
n	18	10	10	9	8
geo Mean [CP]	20,70	31,39	31,02	29,80	34,12
ar Mean [CP]	20,72	31,47	31,36	29,87	34,16
min [CP]	19,63	28,01	24,87	26,78	31,93
max [CP]	22,85	35,57	40,00	32,81	36,33
std dev [± CP]	0,72	2,01	4,33	1,79	1,48
CV [% CP]	3,47	6,39	13,80	6,01	4,32
min [x-fold]	-2,10	-10,40	-71,02	-8,11	-4,57
max [x-fold]	4,43	18,14	506,04	8,09	4,62
std dev [± x-fold]	1,65	4,03	20,09	3,47	2,78

Figure S1. Dose-response curves of *cyp19a1* (left panel) and *slco1f4* (right panel) for zebrafish embryos exposed to E2 and TES, respectively from 48 to 120 hpf.

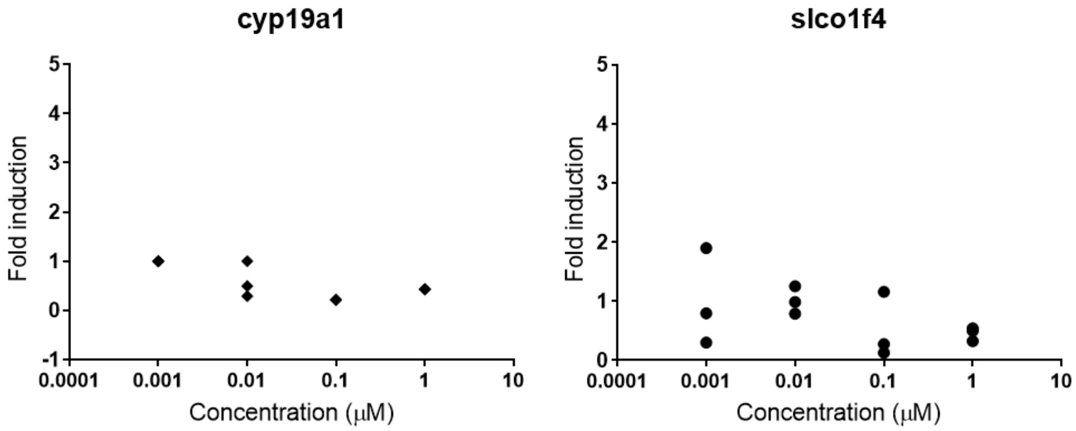

Figure S2. Schematic representation of the main compartments within the HPT axis, and location of the four thyroid markers finally selected (green marks) and the markers not further considered (red marks) in this study. Adapted from [1].

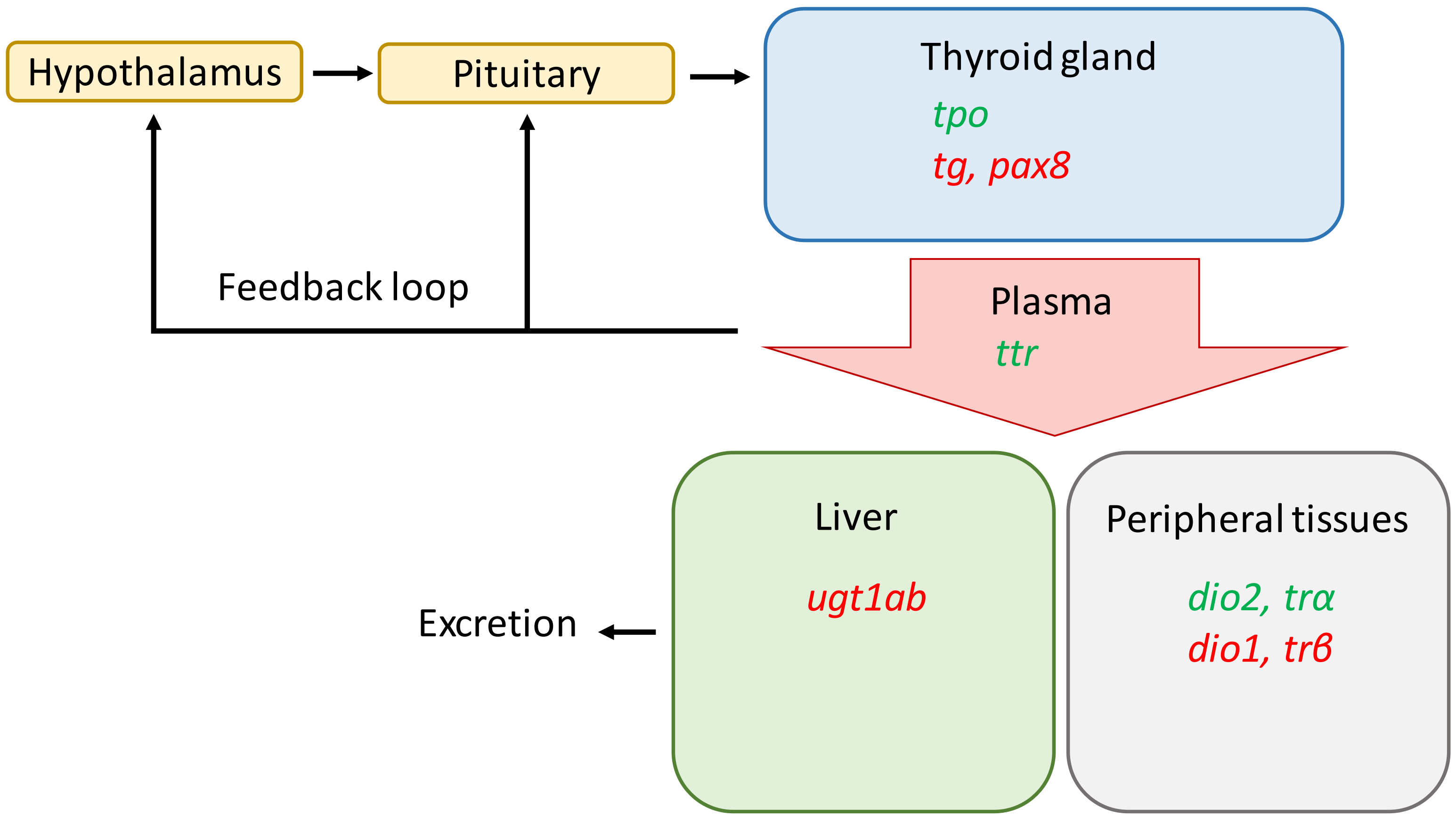

Table S4. Studies evaluated to compare EC50s.

Compound	EC50 (μM)	LogEC50	Exposure phase	endpoint	Reference
E2	0.09	-1.04575749	48-120 h	cyp19a1b mRNA	our study
E2	0.0034	-2.46852108	0-96 h	cyp19a1b - fluorescence	[2]
E2	0.0024	-2.61978876	0-96 h	cyp19a1b - fluorescence	[3]
E2	0.0041	-2.38721614	0-96 h	cyp19a1b - mRNA	[3]
E2	0.01*	-2	0-72 h	cyp19a1b - mRNA	[4]
E2	0.0055*	-2.25963731	72-96 h	cyp19a1b - mRNA	[5]
E2	0.01*	-2	2-48 h	cyp19a1b - mRNA	[6]
E2	n.e.	-	7 days (adults)	cyp19a1b - mRNA	[7]
E2	n.e.	-	21 days (adults)	cyp19a1b - mRNA	[8]
E2	0.0004	-3.39794000	14 days (adults) M[a]	cyp19a1b - mRNA	[9]
E2	0.19	-0.7212464	48-120 h	vtg1 mRNA	our study
E2	0.18*	-0.75696195	0-168 h	vtg1 - mRNA	[10]
E2	0.03*	-1.52287875	0-120 h	vtg1 - mRNA	[11]
E2	0.0002	-3.69897	8 days (adults)	vtg - blood	[12]
E2	0.0006	-3.22184875	24 days (adults)	vtg - blood	[13]
E2	0.00009	-4.04575749	14 days (adults)	vtg - blood	[14]
E2	0.00009	-4.04575749	21 days (adults)	vtg - blood	[15]
E2	0.0006*	-3.22184875	21 days (adults)	vtg - blood	[16]
E2	0.00055*	-3.259637311	21 days (adults)	vtg1 - fluorescence	[17]
BPA	4.99	0.69810055	48-120 h	cyp19a1b - mRNA	our study
BPA	7.4	0.86923172	0-96 h	cyp19a1b - fluorescence	[2]
BPA	3.3	0.51851394	0-96 h	cyp19a1b - fluorescence	[3]
BPA	6.25*	0.79588002	0-96 h	cyp19a1b - fluorescence	[18]
BPA	1.23	0.08990511	0-120 h	ERE-GFP - fluorescence	[19]
BPA	0.22	-0.65757732	0-72 h	cyp19a1b - mRNA	[20]
BPA	3*	0.47712125	72-96 h	cyp19a1b - mRNA	[5]
BPA	0.024*	-1.61978876	14 days (adults)	cyp19a1b - mRNA	[21]
BPA	n.e.	-	48-120 h	vtg1 - mRNA	our study
BPA	n.e.	-	0-168 h	vtg1 - mRNA	[10]
BPA	0.65	-0.18708664	14 days (adults)	vtg - blood	[14]
BPA	0.24*	-0.61978876	14 days (juveniles)	vtg - mRNA	[21]
BPA	2.63*	0.41995575	28 days (juveniles)	vtg - blood	[13]
BPA	1.75*	0.24303805	43 days (adults)	vtg - blood	[22]
BPA	4.93	0.64246452	21 days (adults)	vtg1 - fluorescence	[17]
DES	0.01	-2	48-120 h	cyp19a1b mRNA	our study
DES	0.00001	-5	0-96 h	cyp19a1b - fluorescence	[3]
DES	0.0055*	-2.25963731	2-48 h	cyp19a1b - mRNA	[6]
DES	0.05	-1.30103	48-120 h	vtg1 mRNA	our study
DES	0.03*	-1.52287875	0-168 h	vtg1 - mRNA	[10]
DES	0.0011*	-2.95860731	21 days (juveniles)	vtg - mRNA	[23]
DES	0.0004*	-3.39794000	30 days (adults)	vtg1 - mRNA	[24]
END	n.e.	-	48-120 h	cyp19a1b - vtg1 mRNA	our study

END	n.e.	-	0-96 h	cyp19a1b - fluorescence	[3]
END	0.33	-0.4814860	48-120 h	vtg1 mRNA	our study
END	1.96	0.29225607	6-96 h	vtg mRNA	[25]
END	0.00003	-4.30102999	21 days	vtg - Elisa	[26]
TES	1.11	0.04532298	48-120 h	cyp19a1b - vtg1 mRNA	our study
TES	1.03	0.01283722	0-96 h	cyp19a1b - fluorescence	[3]
TES	1*	0	0-72 h	cyp19a1b - mRNA	[27]
17α-MT	0.62	-0.20760831	48-120 h	cyp19a1b mRNA	our study
17α-MT	0.04	-1.39794001	0-96 h	cyp19a1b - fluorescence	[3]
17α-MT	0.17*	-0.76955108	0-144h	cyp19a1b - mRNA	[28]
17α-MT	2	0.301029996	48-120 h	vtg1 mRNA	our study
17α-MT	0.002*	-2.69897000	21 days (adults)	vtg1 - blood	[29]
17α-MT	0.66	-0.18045606	12 days (adults)	vtg - blood	[30]
TES	0.44	-0.35654732	48-120 h	sult2st3 - cyp2k22 mRNA	our study
TES	0.06	-1.22184875	96-120 h	sult2st3 - cyp2k22 mRNA	[31]
MMI	397	2.59879051	48-120 h	tpo - mRNA	our study
MMI	487	2.68752896	48-120 h	tpo - mRNA	[32]
HEX	2.22	0.34635297	48-120 h	dio2 - mRNA	our study
HEX	3.98*	0.59988307	0-120 h	dio2 - mRNA	[33]

* indicates the mean of an approximate range because authors did not provide specific EC50. n.e. indicates no positive effect. [a] effects only detected in males.

References

1. Jarque S, Piña B. 2014. Deiodinases and thyroid metabolism disruption in teleost fish. *Environ. Res.* 135:361–375.

2. Petersen K, Fetter E, Kah O, Brion F, Scholz S, Tollefsen KE. 2013. Transgenic (cyp19a1b-GFP) zebrafish embryos as a tool for assessing combined effects of oestrogenic chemicals. *Aquat. Toxicol.* 138–139:88–97.

3. Brion F, Le Page Y, Piccini B, Cardoso O, Tong S-K, Chung B, Kah O. 2012. Screening Estrogenic Activities of Chemicals or Mixtures In Vivo Using Transgenic (cyp19a1b-GFP) Zebrafish Embryos. In Vaudry, H, ed., *PLoS One.* 7:e36069.

4. Lassiter CS, Linney E. 2007. Embryonic Expression And Steroid Regulation of Brain Aromatase cyp19a1b in Zebrafish (Danio Rerio). *Zebrafish.* 4:49–58.

5. Chung E, Genco MC, Megrelis L, Ruderman J V. 2011. Effects of bisphenol A and triclocarban on brain-specific expression of aromatase in early zebrafish embryos. *Proc. Natl. Acad. Sci. U. S. A.* 108:17732–7.

6. Kishida M, McLellan M, Miranda JA, Callard G V. 2001. Estrogen and xenoestrogens upregulate the brain aromatase isoform (P450aromB) and perturb markers of early development in zebrafish (Danio rerio). *Comp. Biochem. Physiol. B. Biochem. Mol. Biol.* 129:261–8.

7. Hinfray N, Palluel O, Turies C, Cousin C, Porcher JM, Brion F. 2006. Brain and

gonadal aromatase as potential targets of endocrine disrupting chemicals in a model species, the zebrafish (Danio rerio). *Environ. Toxicol.* 21:332–337.

8. Kallivretaki E, Eggen R, Neuhauss S, Alberti M, Kausch U, Segner H. 2006. Aromatase in zebrafish: A potential target for endocrine disrupting chemicals. *Mar. Environ. Res.* 62:S187–S190.

9. Halm S, Pounds N, Maddix S, Rand-Weaver M, Sumpter J., Hutchinson T., Tyler C. 2002. Exposure to exogenous 17β-oestradiol disrupts P450aromB mRNA expression in the brain and gonad of adult fathead minnows (Pimephales promelas). *Aquat. Toxicol.* 60:285–299.

10. Chen M, Zhang J, Pang S, Wang C, Wang L, Sun Y, Song M, Liang Y. 2018. Evaluating estrogenic and anti-estrogenic effect of endocrine disrupting chemicals (EDCs) by zebrafish (Danio rerio) embryo-based vitellogenin 1 (vtg1) mRNA expression. *Comp. Biochem. Physiol. Part C Toxicol. Pharmacol.* 204:45–50.

11. Muncke J, Eggen RIL. 2006. Vitellogenin 1 mRNA as an early molecular biomarker for endocrine disruption in developing zebrafish (Danio rerio). *Environ. Toxicol. Chem.* 25:2734.

12. Rose J, Holbech H, Lindholst C, Nørum U, Povlsen A, Korsgaard B, Bjerregaard P. 2002. Vitellogenin induction by 17beta-estradiol and 17alpha-ethinylestradiol in male zebrafish (Danio rerio). *Comp. Biochem. Physiol. C. Toxicol. Pharmacol.* 131:531–9.

13. Van den Belt K, Berckmans P, Vangenechten C, Verheyen R, Witters H. 2004. Comparative study on the in vitro/in vivo estrogenic potencies of 17β-estradiol, estrone, 17α-ethynylestradiol and nonylphenol. *Aquat. Toxicol.* 66:183–195.

14. Brian J V., Harris CA, Scholze M, Backhaus T, Booy P, Lamoree M, Pojana G, Jonkers N, Runnalls T, Bonfà A, Marcomini A, Sumpter JP. 2005. Accurate Prediction of the Response of Freshwater Fish to a Mixture of Estrogenic Chemicals. *Environ. Health Perspect.* 113:721–728.

15. Dammann AA, Shappell NW, Bartell SE, Schoenfuss HL. 2011. Comparing biological effects and potencies of estrone and 17β-estradiol in mature fathead minnows, Pimephales promelas. *Aquat. Toxicol.* 105:559–568.

16. Parks LG, Cheek AO, Denslow ND, Heppell SA, McLachlan JA, LeBlanc GA, Sullivan C V. 1999. Fathead minnow (Pimephales promelas) vitellogenin: purification, characterization and quantitative immunoassay for the detection of estrogenic compounds. *Comp. Biochem. Physiol. Part C Pharmacol. Toxicol. Endocrinol.* 123:113–125.

17. Zhiqiang Zeng, Tao Shan, Yan Tong, Siew Hong Lam and, Gong* Z. 2005. Development of Estrogen-Responsive Transgenic Medaka for Environmental Monitoring of Endocrine Disrupters. doi:10.1021/ES050728L.

18. Le Fol V, Aït-Aïssa S, Sonavane M, Porcher J-M, Balaguer P, Cravedi J-P, Zalko D, Brion F. 2017. In vitro and in vivo estrogenic activity of BPA, BPF and BPS in zebrafish-specific assays. *Ecotoxicol. Environ. Saf.* 142:150–156.

19. Green JM, Metz J, Lee O, Trznadel M, Takesono A, Brown AR, Owen SF, Kudoh T, Tyler CR. 2016. High-Content and Semi-Automated Quantification of Responses to Estrogenic Chemicals Using a Novel Translucent Transgenic Zebrafish. *Environ. Sci. Technol.* 50:6536–6545.

20. Saeed A, Hashmi I, Zare A, Mehrabani-Zeinabad M, Achari G, Habibi HR. 2016. Efficacy of UV-C photolysis of bisphenol A on transcriptome alterations of genes in zebrafish embryos. *J. Environ. Sci. Heal. Part A.* 51:877–883.

21. Molina A, Abril N, Morales-Prieto N, Monterde J, Ayala N, Lora A, Moyano R. 2018. Hypothalamic-pituitary-ovarian axis perturbation in the basis of bisphenol A (BPA) reproductive toxicity in female zebrafish (Danio rerio). *Ecotoxicol. Environ. Saf.* 156:116–124.

22. P. Sohoni †, C. R. Tyler *,‡, K. Hurd §, J. Caunter §, M. Hetheridge §, T. Williams §, C. Woods §, M. Evans §, R. Toy ∥, M. Gargas ⊥ and, Sumpter† JP. 2001. Reproductive Effects of Long-Term Exposure to Bisphenol A in the Fathead Minnow (Pimephales promelas). doi:10.1021/ES000198N.

23. Zhong X, Xu Y, Liang Y, Liao T, Wang J. 2004. Vitellogenin in rare minnow (Gobiocypris rarus): identification and induction by waterborne diethylstilbestrol. *Comp. Biochem. Physiol. Part C Toxicol. Pharmacol.* 137:291–298.

24. Yin P, Li Y-W, Chen Q-L, Liu Z-H. 2017. Diethylstilbestrol, flutamide and their combination impaired the spermatogenesis of male adult zebrafish through disrupting HPG axis, meiosis and apoptosis. *Aquat. Toxicol.* 185:129–137.

25. Moon Y-S, Jeon H-J, Nam T-H, Choi S-D, Park B-J, Ok YS, Lee S-E. 2016. Acute toxicity and gene responses induced by endosulfan in zebrafish (Danio rerio) embryos. *Chem. Speciat. Bioavailab.* 28:103–109.

26. Han Z, Jiao S, Kong D, Shan Z, Zhang X. 2011. Effects of β-endosulfan on the growth and reproduction of zebrafish (Danio rerio). *Environ. Toxicol. Chem.* 30:2525–2531.

27. Mouriec K, Gueguen M-M, Manuel C, Percevault F, Thieulant M-L, Pakdel F, Kah O. 2009. Androgens Upregulate cyp19a1b (Aromatase B) Gene Expression in the Brain of Zebrafish (Danio rerio) Through Estrogen Receptors1. *Biol. Reprod.* 80:889–896.

28. Trant JM, Gavasso S, Ackers J, Chung BC, Place AR. 2001. Developmental expression of cytochrome P450 aromatase genes (CYP19a and CYP19b) in zebrafish fry (Danio rerio). *J. Exp. Zool.* 290:475–83.

29. Pawlowski S, Sauer A, Shears J., Tyler C., Braunbeck T. 2004. Androgenic and estrogenic effects of the synthetic androgen 17α-methyltestosterone on sexual development and reproductive performance in the fathead minnow (Pimephales promelas) determined using the gonadal recrudescence assay. *Aquat. Toxicol.* 68:277–291.

30. Ankley GT, Jensen KM, Kahl MD, Korte JJ, Makynen EA. 2001. Description and evaluation of a short-term reproduction test with the fathead minnow

(Pimephales promelas). *Environ. Toxicol. Chem.* 20:1276–1290.

31. Fetter E, Smetanová S, Baldauf L, Lidzba A, Altenburger R, Schüttler A, Scholz S. 2015. Identification and Characterization of Androgen-Responsive Genes in Zebrafish Embryos. *Environ. Sci. Technol.* 49:11789–98.

32. Fetter E, Baldauf L, Da Fonte DF, Ortmann J, Scholz S. 2015. Comparative analysis of goitrogenic effects of phenylthiourea and methimazole in zebrafish embryos. *Reprod. Toxicol.* 57:10–20.

33. Yu L, Chen M, Liu Y, Gui W, Zhu G. 2013. Thyroid endocrine disruption in zebrafish larvae following exposure to hexaconazole and tebuconazole. *Aquat. Toxicol.* 138–139C:35–42.
